# Impairments of Sociocognitive Functions in Individuals with Behavioral Addictions: A Review Article

**DOI:** 10.1007/s10899-023-10227-w

**Published:** 2023-06-12

**Authors:** Dalia Arafat, Patrizia Thoma

**Affiliations:** Neuropsychological Therapy Centre, Faculty of Psychology, Ruhr University Bochum, Bochum, Germany

**Keywords:** Emotion recognition, Social cognition, Empathy, Theory of mind, Behavioral addiction

## Abstract

Since little is known about the exact pattern of social cognitive impairments related to behavioral addictions, the aim of the PRISMA-oriented review was to (i) provide an overview of relevant empirical publications, and to (ii) to elucidate which specific aspects of social cognition (i.e., emotion recognition, empathy, and theory of mind (ToM)) are impaired in different types of behavioral addictions. Behavioral addictions have been associated with cognitive deficits which may contribute to impaired social cognitive functioning. More recently, this domain has been investigated in patients with behavioral addictions as impaired social cognition detrimentally affects daily functioning and thus forms a relevant target for treatment. A systematic search in the PubMed and Web of Science databases was performed focusing on social cognitive functions in behavioral addictions. Studies focusing on the same social cognitive component were grouped together, this was done based on the used assessment measures. In total, 18 studies met the specified inclusion criteria. Five studies focusing on emotion recognition concluded that individuals with behavioral addictions show impairments in this domain. As for the 13 studies focusing on empathy and/or ToM, most of them found deficits linked to different types of behavioral addictions. Only two studies, one of which was investigating a distinct population (multiplayer online role-playing gamers) did not link empathy to behavioral addictions. The results show that the majority of studies focusing on social cognition and behavioral addictions found some deficits. Additional research focusing on this topic is urgently needed in behavioral addictions, addressing several methodological issues.

**Supplementary Information:**

The online version contains supplementary material available at 10.1007/s10899-023-10227-w.

## Introduction

The term “addiction” is commonly used when referring to Substance Use Disorders (SUD), which are mainly characterized by loss of control over one’s substance use in spite of its detrimental consequences, tolerance effects entailing a need for consumption of higher quantities of the substance of abuse in order to achieve the same effects and withdrawal symptoms when substance use is prevented or delayed (Zou et al., 2017). The term behavioral addictions refers to non-substance addictions, often exhibiting similar features as SUD (e.g., loss of control, tolerance, and withdrawal) (Alavi et al., 2012) (see below for a detailed definitions). Behavioral addictions can also be defined as a compulsive engagement in a certain activity that does not involve substance intake in spite of its negative physical, mental, social, and/or financial consequences (Chen et al., 2017a). Prominent instances of behavioral addictions are Gambling Disorder and Internet Gaming Disorder. The nucleus accumbens along with other limbic brain structures, such as the amygdala, the anterior cingulate cortex, and the hippocampus, is relevant for both SUD (Koob & Volkow, 2016) and behavioral addictions (Quintero Garzola, 2019). Several studies have found that there are structural brain differences in participants with behavioral addictions when compared to healthy individuals, such as changes in gray and white matter in several areas of the brain (Mohammadi et al., 2020).

Similar to the comorbidities related to SUD (Common Comorbidities with Substance Use Disorders Research Report, 2020), there is also an association between behavioral addictions and different psychological disorders, such as Depression, Anxiety, and Personality Disorders (e.g. Borderline Personality Disorder and Antisocial Personality Disorder: Brown et al., 2015; Lorains et al., 2011) among others (King et al., 2013; Matar Boumosleh & Jaalouk, 2017; Pietrzak et al., 2007; Vaddiparti & Cottler, 2017). Psychiatric conditions, in turn, are known to affect the performance in various domains of social cognition (Bora & Berk, 2016; Langarita-Llorente & Gracia-Garcia, 2019).

Social cognition allows us to navigate the social world and to adequately perceive, process and respond to social information (Kennedy & Adolphs, 2012) (see detailed definition below). Several brain structures are considered important structures implicated in social cognition such as the amygdala, the anterior cingulate and the hippocampus, which also, as previously mentioned, are relevant in behavioral addictions (Koban & Pourtois, 2014; Tso et al., 2018).

Social cognition is known to be impaired in both adults and adolescents with SUD (e.g. Thoma et al., 2013; Winters et al., 2021). However, until now, little is known about the exact pattern of impairments in different domains of social cognition in individuals suffering from behavioral addictions. The aim of the current PRISMA-oriented review is to provide a systematic overview of research on social cognition in different types of behavioral addictions, to pinpoint the patterns of sociocognitive impairment related to behavioral addictions and to outline possible clinical implications.

This review is structured as follows: First, relevant terms will be defined: behavioral addictions, social cognition in general and the relevant subdomains of emotion recognition, empathy, and Theory of Mind (ToM), that will be focused on in this review. After that, the literature which investigated social cognition in different types of behavioral addictions will be systematically reviewed. At the end, directions for further research will be outlined.

### Definition of Relevant Terms: Behavioral Addictions

Behavioral addictions have only recently sparked increasing research interest, although it is, overall, not a new concept. Goodman (1990) proposed a diagnostic criteria for behavioral addictions as shown in Table 1.

**Table 1:** Diagnostic criteria for behavioral addictions (Goodman, 1990)

A. Recurrent failure to resist impulses to engage in a specified behavior
B. Increasing sense of tension immediately prior to initiating the behavior
C. Pleasure or relief at the time of engaging in the behavior
D. A feeling of lack of control while engaging in the behavior
E. At least five of the following: (1) frequent preoccupation with the behavior or with activity that is preparatory to the behavior (2) frequent engaging in the behavior to a greater extent or over a longer period than intended (3) repeated efforts to reduce, control or stop the behavior (4) a great deal of time spent in activities necessary for the behavior, engaging in the behavior or recovering from its effects (5) frequent engaging in the behavior when expected to fulfill occupational, academic, domestic or social obligations (6) important social, occupational or recreational activities given up or reduced because of the behavior (7) continuation of the behavior despite knowledge of having a persistent or recurrent social, financial, psychological or physical problem that is caused or exacerbated by the behavior (8) tolerance: need to increase the intensity or frequency of the behavior in order to achieve the desired effect or diminished effect with continued behavior of the same intensity (9) restlessness or irritability if unable to engage in the behavior
F. Some symptoms of the disturbance have persisted for at least 1 month, or have occurred repeatedly over a longer period of time

As yet, specific and uniform official diagnostic criteria are not available for each subtype of behavioral addictions (Zou et al., 2017), which also share many common symptoms (Grüsser & Thalemann, 2006). To start with gambling, both the DSM-5 (American Psychiatric Association, 2013) and the ICD-11 (World Health Organization, 2019) consider persistent and recurrent problematic gambling an addictive disorder. Gambling Disorder shows an estimated worldwide prevalence of 0,1% to 0,7% (see Petry et al., 2018). Internet Gaming Disorder is mentioned as a condition with proposed criteria for further study (section III) in the DSM-5 (American Psychiatric Association, 2013) and can include offline computerized games as a subtype. In the ICD-11, the World Health Organization (World Health Organization, 2018) also recognized a persistent or recurrent Gaming Disorder (online and offline) as a new mental health condition. Excessive gambling online may qualify for a separate diagnosis of Gambling Disorder or Internet Gaming Disorder. Estimated prevalence rates for Internet Gaming Disorder vary between 0.3 and 4.9%, with the wide range being also partly due to the lack of established diagnostic criteria and diagnostic instruments with adequate psychometric properties (see Petry et al., 2018).

Gambling Disorder is the first recognized behavioral addiction disorder which has established diagnostic criteria according to the DSM-5. Four out of nine criteria, largely resembling the previously outlined general criteria for behavioral addictions proposed by Goodman (1990) have to be met within a period of one year: thinking about gambling excessively (i.e., “pre-occupation”); betting greater amounts (i.e., tolerance); being unable to cease or reduce gambling; exhibiting withdrawal symptoms (restlessness, irritability) when not gambling; gambling to escape adverse moods or problems; attempting to win back losses (i.e., “chasing”); financially relying on others to cover losses; lying about or covering up gambling; and losing important relationships, or a career or educational opportunity, because of gambling. Several structured interviews have been developed for the assessment of the DSM-5 criteria, such as the National Opinion Research Centre Screen (Gerstein & Toce, 1999).

For Internet Gaming Disorder, the DSM-5 lists nine possible criteria and it is proposed to use meeting five criteria as a cut-off for diagnosis: pre-occupation with games; tolerance toward games or gaming; inability to cease or reduce gaming; withdrawal symptoms when gaming is not possible; gaming to escape adverse moods or problems; loss of interests in other activities; continued excessive gaming despite knowledge of problems; lying about or covering up gaming; and risking or losing a relationship, job, or vocational or education opportunity because of gaming. Internet Gaming Disorder is not well explored in clinical samples when compared to Gambling Disorder and still needs further research to be recognized as a unique addictive disorder by the American Psychiatric Association. Both gambling and Internet Gaming Disorders are characterized by impaired control over the addictive behavior, increasing priority over other interests, continuation/escalation despite negative consequences and significant distress or impairment in important areas of functioning (American Psychiatric Association, 2013; World Health Organization, 2018). Although gambling and gaming thus overlap to some degree, there are two main differences between the two: Gambling revolves around risking real money and expecting larger amounts in return, which is usually not the case in gaming. Gaming is also largely skill and/or knowledge dependent while only some forms of gambling could partially be skill dependent (e.g. card games) (Petry et al., 2018).

For excessive internet use or internet addiction respectively, which does not involve playing online games, no diagnostic criteria have been established as yet, since it is considered a new phenomenon and needs further research (Zegarra Zamalloa & Cuba Fuentes, 2017) However, in studies focusing on internet addiction, a questionnaire by Young (1998) is commonly used as a diagnostic tool. It is based on the diagnostic criteria for pathological Gambling Disorder as specified in the DSM-4, adapted for the assessment of internet addiction.

Exploring different behavioral addictions to fill the gaps for each type of internet addiction is important as excessive pornography viewing could be different from excessive social network sites (SNS) use. Additionally, paying attention to whether the behavioral addiction is leading to substantial distress and impairment in everyday life is crucial to delineate it from a mental disorder, which would not apply to, e.g., spending time on the internet instead of doing house chores, or to frequently checking one’s smartphone/watching TV rather than going to bed (Petry et al., 2018).

With the excessive use that might arise from the availability of the internet and different social platforms nowadays, in the present review, the studies which focused on internet, SNS, and video gaming addiction were included along with gambling addiction.

There are other types of behavioral addictions which were not included in the current review such as Food Addiction, as it may potentially be better classified under a different category as opposed to addictive disorders, such as Feeding and Eating Disorders. Pyromania (fire-setting) and Kleptomania (stealing) are not considered addictive behaviors, they are considered as Disruptive, Impulse Control, and Conduct Disorders in DSM-5, and as Impulse Control Disorders in ICD-11. According to the ICD-11, Compulsive Sexual Behavior Disorder and Compulsive Buying-Shopping Disorder are also considered as Impulse Control Disorders and not as an addiction, but neither is considered as a disorder in the DSM-5 due to lack of evidence. The same also applies to e.g. exercise addiction which is not considered as an addiction disorder (American Psychiatric Association, 2013, World Health Organization, 2019).

### Definition of Relevant Terms: Social Cognition

Social cognition is a broad term that can be defined as any cognitive or emotional process that is involved in one-to-one or group social interactions (Frith & Blakemore, 2006). Social cognition partly also relies on more general cognitive processing, such as memory and executive functioning (McKinnon & Moscovitch, 2007). It encompasses abilities which guide our social behavior through constructing and using representations of the relations between oneself and others (Adolphs, 2001). Among the most relevant social cognition subdomains are emotion recognition, empathy and ToM, which will be focused on in this review and elaborated on in the respective definitions.

#### Emotion Recognition

Decoding facial expressions is of primordial importance during everyday communication promoting socially appropriate responses (Argaud et al., 2018), but is also complemented by inferring other people’s emotions from voices (prosody) (Lausen & Hammerschmidt, 2020) and their body postures (de Gelder et al., 2015). Ekman and Cordaro (2011) named the following universal emotions: anger, fear, surprise, sadness, disgust, contempt, and happiness. In the current review, the retrieved studies assessed most of these emotions in addition to more complex ones (peacefulness, threat).

#### Empathy and ToM

One further essential component for successful social interactions is empathy (Decety, 2011). Empathy may involve experiencing a shared emotional state as a result of perceiving another person’s emotions or responding to the emotional situation of another individual on an affective level, but with preserving self-other distinction (Preston & de Waal, 2002). Usually, two components of empathy are distinguished: The cognitive empathy component is similar to ToM as it is related to understanding what emotions another person is feeling. The emotional empathy component is the ability to share and resonate with another person’s feelings (de Vignemont & Singer, 2006; Wilson et al., 2017) or to affectively respond to them. The affective response to another person’s emotional state can be self-oriented (e.g. personal distress in response to another person’s negative emotions) or reflect empathic concern in terms of an other-oriented emotion (Batson, 2018; Decety & Lamm, 2006).

Another sociocognitive ability which is more cognitive than emotional is ToM, also known as mentalizing denoting “the ability to understand what other people want, think, believe”. ToM is a complex construct that again can be divided into a cognitive and affective component, i.e., a cognitive understanding of the beliefs, intentions and thoughts of other people and having assumptions about other people’s emotional state (Shamay-Tsoory & Aharon-Peretz, 2007). This also includes inferring mental states which are not directly observable, all of which facilitates the prediction of other people’s behaviors (Premack & Woodruff, 1978). Empathy and ToM partly overlap when it comes to cognitively inferring another person’s emotional states (cognitive empathy/affective ToM) (e.g. Shamay-Tsoory, 2011).

Although in the retrieved literature, the terms empathy and ToM are sometimes used interchangeably, within this review, social cognition will be used as the overarching term encompassing emotion recognition, empathy, and ToM as distinct subconcepts. When referring to empathy, it includes understanding the emotional state and the ability to produce the appropriate emotional response. When speaking of ToM, it involves the understanding of intentions, beliefs, desires, thoughts, and understanding the other person’s experience.

### Aims and Scope of the Present Review

SUD are often associated both with sociocognitive problems in the domains of empathy and ToM (Massey et al., 2018; Sanvicente-Vieira et al., 2017; Thoma et al., 2013) and with cognitive impairment affecting memory, attention, and executive functions (Curran et al., 2016; Hoffman et al., 2006; Indlekofer et al., 2009). Cognitive deficits can be seen in individuals with behavioral addictions (Brand et al., 2005; Dong et al., 2011; Zhou et al., 2014), which may also contribute to impaired social cognitive functioning Channon, 2004; Thoma et al., 2013).

It has also been proposed that there is a link between poor social cognition and addiction, however, the nature of the relationship is still unclear, e.g. regarding the issue whether ToM impairment is a consequence of the substance use or the other way round (Sanvicente-Vieira et al., 2017). Impaired social cognition can also affect social integration negatively in addicted patients (Volkow et al., 2011), thus it is an important construct to look into.

In recent years, interest in research that focuses on patients with different types of addictions and their sociocognitive abilities has been increasing. Also, as previously mentioned, new types of addiction emerged. There are only two identified reviews that dealt with behavioral addictions and social cognition, one was published by Hurel et al. (2019). This review focused on gambling, and only one study matched the specified inclusion criteria. This study focused on gambling and social cognition (Kornreich et al., 2016) and was the first study focusing on a purely behavioral addiction. It yielded evidence of non-verbal perception deficits in this kind of sample assessing musical, vocal and facial emotion detection and intensity. The other review was by Wu et al. (2022), in which authors focused on empathy and gambling behaviors both in healthy individuals, where non-addicted populations were investigated in laboratory settings while performing gambling tasks such as wheels of fortune, and also in individuals with Gambling Disorder. Only one study focusing on Gambling Disorder was included in their review (Tomei et al., 2017) which was also retrieved in the systematic search for the current review. The study focused on self-reported empathy in Gambling Disorder and will be discussed in the designated section for empathy.

In the present review, we aim to systematically review current empirical publications that focused on different types of behavioral addictions in association with functioning in at least one of the previously mentioned three subdomains of sociocognitive functioning: emotion recognition, empathy, and ToM. Sociocognitive impairments often entail a higher relapse risk in SUD (Le Berre & Le Berre, 2019). Due to a small number of studies in the behavioral addictions field as opposed to SUD, the current review of the available data can lead to a better understanding of sociocognitive impairment in individuals with different types of behavioral addictions. This could promote the development of more effective interventions integrating sociocognitive aspects into the treatment plan, e.g. by including social skills training as part of cognitive therapy (Sylvian et al., 1997). Developing new treatments based on results from this review could lead to a lower relapse rate, as it has been found that, similarly to the situation in SUD, some neurocognitive deficits are also strong predictors of relapse in behavioral addictions (Goudriaan et al., 2008).

## Method

### Search Criteria

A systematic search of the PubMed and Web of Science databases was performed using the following search terms: “gambl*” OR “gaming” OR “game” OR “internet” OR “social network sites” OR “social media” OR “SNS” in combination with “social cognition” OR “empat*” OR “ToM” OR “theory or mind” OR “mentalizing” OR “emotion recognition” OR “social skills” OR “social problem solving” OR “emotion recognition” OR “affect recognition” in abstract OR title in both databases. The search syntaxes for each database are presented as supplementary material (file 1).

Additionally, the reference lists of the retrieved articles were screened for additional eligible studies. The screening and evaluation of the full texts were carried out independently by the first author (DA), in several cases the co-author (PT) was requested for an appraisal for ambiguous cases. Some records were excluded using automation tools available in the database search. The following filters were used for PubMed and Web of Science searches respectively: English, Humans/ documents types: articles and review articles, English language, research areas: psychology or psychiatry or neuroscience. An initial literature search was conducted in December 2021 and an update was carried out in June 2022, thus all articles published until June 2022 were searched. Eligibility of articles was determined using a two-stage screening process consisting of (1) manual title and abstract screening, and (2) full-text review. When screening the title and abstract was not sufficient to reach an initial decision about inclusion, the entire article was reviewed. Studies focusing on non-adult (< 18 years of age) samples were excluded in addition to studies focusing on short-term effects, such as experiments where participants were asked to play games in an experimentally controlled setting such as in a laboratory (e.g. Jerabeck & Ferguson, 2013) or at home (e.g. Kühn et al., 2019) with a certain frequency and for a short period of time, or when habitual players were asked about their gaming frequency without measuring addictive symptoms (e.g. Pichon et al., 2021). Only studies that involved clinically diagnosed participants with behavioral addictions, or that measured excessive or problematic symptoms of behavioral addictions were included, if they also assessed sociocognitive functions. In addition to that, studies that used only neuroimaging techniques without reporting any relevant behavioral data were excluded. Investigating sociocognitive impairments in individuals with behavioral addiction who additionally also utilize substances or are also suffering from other (co-morbid) psychiatric disorders were also excluded due to the unclear effects. No further exclusion criteria were set.

**Fig. 1:**
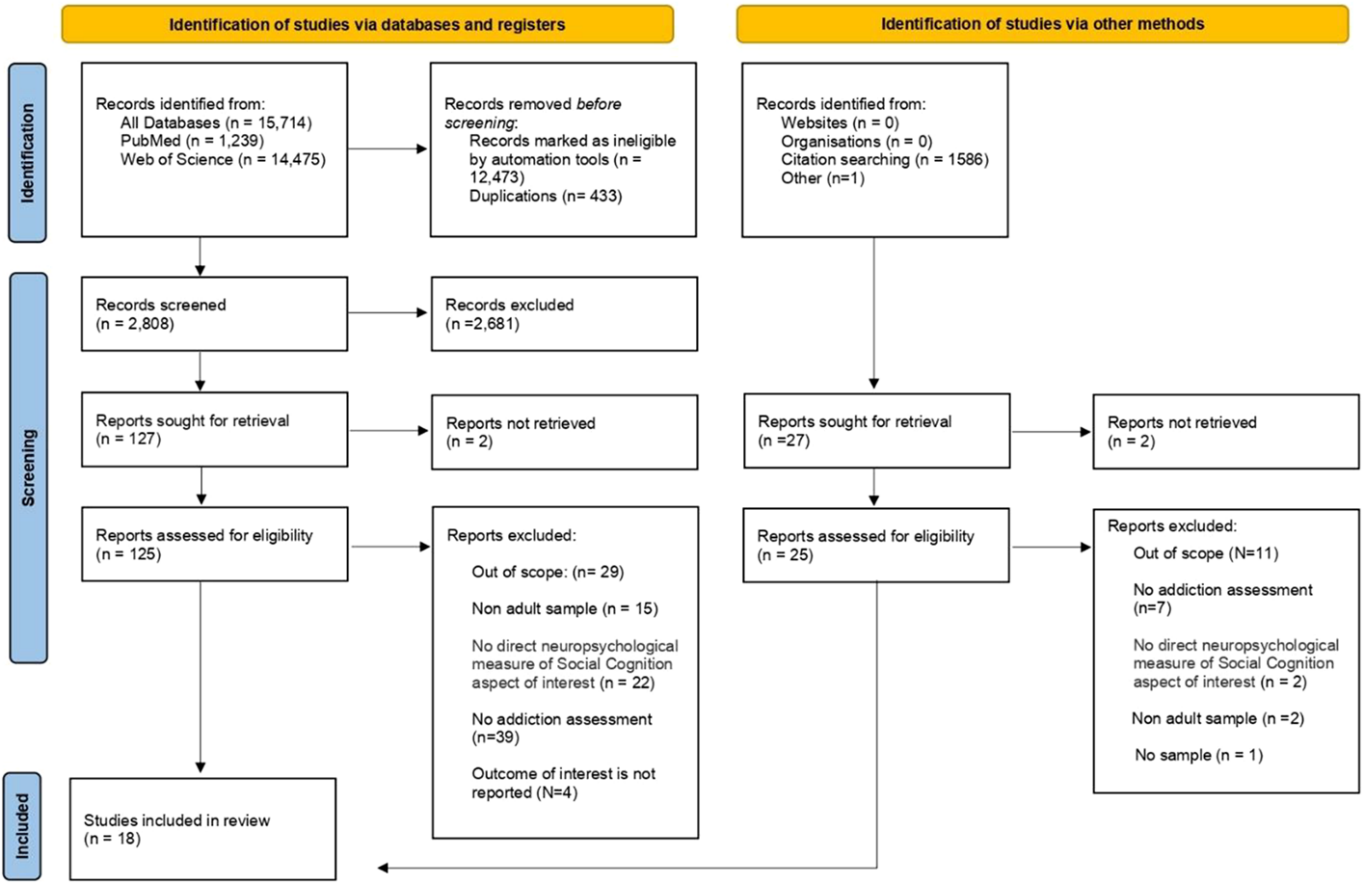
Prisma flow diagram

PRISMA guidelines (Page et al., 2021) served as an orientation for the present review and 16 studies were retrieved applying the automated search. Additionally, using the manual search, the above-mentioned study by Kornreich et al. (2016), which focused on different aspects of social cognition in gambling addiction was included, and another study addressing emotion recognition in Internet Gaming Disorder by Peng et al. (2017). Thus, in total, 18 studies were included in the review grouped according to investigated social cognition subcomponents in the sections below. Figure 1 presents the results of the search and selection process in a flow diagram. The data supporting the results are available in the subsequent paragraphs.

## Results

Five studies were retrieved that focused on emotion recognition, and 13 focused on empathy and/or ToM. The summary table is presented as supplementary material (file 2).

### Emotion Recognition

Five studies (Chen et al., 2017b; Engelberg & Sjöberg, 2004; Fan et al., 2022; Kornreich et al., 2016; Peng et al., 2017) focused on emotion recognition in participants with different forms of behavioral addictions: pathological gambling (n = 1), internet addiction (n = 2), and internet gaming addiction (n = 2), all of which found some deficits in emotion recognition. As studies used different tasks to assess emotion recognition skills, the retrieved studies will be grouped based on the type of behavioral addiction that was focused on.

Kornreich et al. (2016) focused on pathological gamblers who were seeking ambulatory treatment for the gambling problem. The sample consisted of 22 participants with pathological gambling and 22 controls matched for sex (all male), age, and educational level. Participants were diagnosed according to the DSM-4-TR criteria for pathological gambling. They were asked to perform emotion recognition tasks for musical stimuli conveying happiness, sadness, threat, peacefulness (Vieillard et al., 2008), for vocal stimuli conveying anger, disgust, fear, sadness, surprise, happiness, or a neutral mood (Belin et al., 2008) and for faces reflecting happiness, sadness, fear, anger or neutral expressions (Maurage et al., 2007). No significant differences were found between pathological gamblers and healthy controls regarding the recognition accuracy for the musical emotions, however controls and patients differed regarding the vocal and facial modality. The pathological gamblers were overall less accurate in identifying emotions compared to controls, especially for neutral vocal stimuli. Concerning the intensity, the pathological gamblers underestimated the intensity of peacefulness in music and overestimated emotional intensity in neutral voices and faces compared to controls. Anxiety levels, which were assessed using the State Trait Inventory Anxiety by Spielberger (1983), did not explain the observed deficits but seem to have accounted for overestimation of emotions in neutral faces and accuracy problems in detecting fear in voices in pathological gamblers. Another two studies (Fan et al., 2022; Peng et al., 2017) focused on Internet Gaming Disorder measuring gaming addiction using Young’s Internet Addiction Test (1998). Peng et al. (2017) applied an additional inclusion criterion for the gaming group: playing online games ≥ 4 h per day and ≥ 30 h per week. The sample consisted of two groups: 16 gamers and 16 controls, comparable on age, handedness, and education. In the study by Fan et al. (2022), the sample consisted of two groups: 60 participants diagnosed by a psychiatrist with Internet Gaming Disorder and 60 controls. Peng et al. (2017) used a computerized emotion recognition task which encompassed neutral, happy, and sad faces. Fan et al. (2022) administered an emotion recognition task involving happy, neutral and angry faces. In this task, the pictures were morphed, the ears, hair, and neck were removed and then edited to standardize the pixel, brightness, contrast, and resolution.

Although both studies found overall differences between the gaming addiction groups and controls, there were also emotion specific differences. Peng et al. (2017) found that similarly to the control group, the Internet Gaming Disorder group responded faster to happy and sad compared to neutral facial expressions. However, they were slower than controls when responding to sad compared to neutral expressions in the sad block. Concerning accuracy, there were no significant differences in the study by Peng et al (2017) but Fan et al. (2022) found differences in reaction time in addition to accuracy between the groups. The addiction group was less accurate in recognizing happiness compared to anger which could reflect a negative bias in emotion recognition. Peng et al. (2017) also argued that happy and sad expressions are considered unconscious emotional expressions which might already be recognized in the pre-attentive stage that seems unimpaired in internet gamers.

As for the two studies which focused on internet addiction, two used the common assessment tool developed by Young (1998) to assess internet addiction (Chen et al., 2017b; Engelberg & Sjöberg, 2004). Engelberg et al. (2004) assessed 41 participants using emotion recognition tasks based on facial expressions and social episodes taken from an emotion intelligence measure (Mayer et al., 2000). Chen et al., (2017b) used a forced-choice computer task to measure facial expression recognition for the basic emotions (anger, disgust, fear, happiness, sadness, surprise) and their sample consisted of 97 participants (54% no-internet addiction symptom group, 41% mild-internet addiction symptom group, and 5% severe-internet addiction symptom group). Engelberg et al. (2004) found that internet addiction was related negatively to the ability to recognize emotions both in facial expressions and in social episodes. They also found that facial emotion recognition deficits explained more variance for internet addiction than emotion recognition deficits in social episodes. Chen et al., (2017b) on the other hand found emotion specific deficits: The severe- symptom group showed more difficulty in recognizing disgust than the no- and mild-symptom groups, an effect which was also positively associated with both self-perceived stress and the severity of internet addiction. However, the deficits in recognizing disgust disappeared after controlling for the effects of self-perceived stress. According to the authors, disgust is more difficult to recognize than other facial emotions. An impairment in facial disgust recognition during communication could lead to higher chances of experiencing interpersonal conflicts, such as not understanding that one should stop a certain inappropriate behavior, such as internet overuse. On the other hand, having more conflicts could elevate stress levels, which can render it even more difficult to self-regulate and more difficult to control the time spent on the internet.

Taken together, emotion recognition seems to be impaired in different behavioral addictions, although some of these deficits could be mediated by factors other than addiction.

### Empathy and ToM

Several studies focused on empathy (*n* = 10) and/or ToM (*n* = 3) in different types of behavioral addictions: gambling addiction, gaming addiction, internet addiction, smartphone addiction, Facebook addiction, and SNS addiction. In the following section, these studies will be grouped based on the used measures.

#### Empathy

All of the retrieved studies assessing empathy in association with behavioral addiction used self-report measures except for one, which used an empathy for pain paradigm. Kopiś-Posiej et al. (2021) studied empathy in association with problematic Facebook use as assessed by the Facebook Intrusion Scale (Elphinston & Noller, 2011). Participants were grouped according to whether their Facebook use was problematic or not based on their answers on this scale. During task administration, painful and non-painful pictures were shown both in a Facebook context and in a neutral context. The sample consisted of 21 participants in the low problematic Facebook use group and 22 participants in the high problematic Facebook use group. The high problematic Facebook use group responded faster in the neutral context compared to the Facebook context where they spent more time observing Facebook-related stimuli. In both the high and the low problematic Facebook use group, the pain shown in the stimuli presented in the Facebook context was rated as slightly lower than when the stimuli were presented in a neutral context. The authors argue that the context specific reaction time differences may not be connected to empathy. On the contrary, the familiar Facebook layout might have provided a distraction from the stimuli.

The rest of the studies, grouped in this section, used self-report measures. Six retrieved studies used the 16-item Interpersonal Reactivity Index (IRI) (Davis, 1983), which assesses self-rated aspects of emotional (Empathic Concern (EC) and Personal Distress (PD)) and cognitive (Perspective Taking (PT) and Fantasy (FS)) empathy. In terms of the investigated populations, one of the retrieved studies focused on problematic gambling (Tomei et al., 2017), three focused on individuals with internet addiction, and additionally one of these also assessed smartphone addiction (Jiao et al., 2017; Lachmann et al., 2018; Melchers et al., 2015), two on problematic gaming (Cudo et al., 2019; Mohammadi et al., 2020), and one on SNS addiction (Stockdale & Coyne, 2020). Three studies additionally used the Empathy Quotient (EQ) (Baron-Cohen & Wheelwright, 2004), another self-report measure, which assesses self-reported global empathy. It contains 40 empathy items, which gauge both cognitive and affective empathy (emotional reactivity) and social skills, in addition to 20 filler/control items. One of the retrieved studies used the EQ and focused on problematic gaming (Collins & Freeman, 2013), and another two will be first mentioned in the IRI section (Lachmann et al., 2018; Melchers et al., 2015). The last study (Dell’Osso et al., 2019) in this section used the empathy subscale of The Adult Autism Subthreshold Spectrum (AdAS) questionnaire (Dell’Osso et al., 2017), which explores different domains of the wide spectrum of autism manifestation.

##### IRI

Five studies used the IRI (Cudo et al., 2019; Jiao et al., 2017; Mohammadi et al., 2020; Stockdale & Coyne, 2020; Tomei et al., 2017) in gambling, gaming, internet and SNS addictions. Tomei et al. (2017) recruited 21 problem gamblers who were treated for gambling addiction in an outpatient clinic and were assessed using The Problem Gambling Severity Index (Ferris & Wynne, 2001), in addition to 24 healthy gamblers and 31 non-gamblers, all unmatched on several variables. Jiao et al. (2017) used Young’s test (1998) to measure internet addiction and the sample consisted of two groups: 16 participants in the internet addiction group and 16 healthy controls. Mohammadi et al. (2020) focused on violent video gaming and used the Scale for Internet Addiction (Hahn & Jerusalem, 2010) and the sample consisted of 29 gamers playing violent videogames and 29 controls matched regarding age, sex, school education, and handedness. Cudo et al. (2019) assessed problematic video gamers using the Problem Videogame Playing questionnaire (Tejeiro et al., 2016), and the sample consisted of 370 participants (169 male gamers). Stockdale and Coyne (2020) administered a modified version of the Problematic use of Mobile Phones scale (Merlo et al., 2013) to 385 participants in order to assess social media addiction.

For gambling addiction, Tomei et al. (2017) found that problem gamblers scored higher on PD and lower on FS and PT scales compared to non-gamblers (did not gamble during last year) and healthy gamblers (gambled during the last year but did not have gambling problems) taken together as one group. The authors also compared healthy gamblers to non-gamblers, and did not find any significant differences between these two groups.

Jiao et al. (2017) found differences only in IRI PD scores between the control and addiction group. Their results are inconsistent with the previous studies as they found that the internet addiction group scores on IRI PD were lower than in controls. This also indicates an impairment in cognitive processes. The authors backed this conclusion with an investigation using electroencephalography in which the addiction group did not show any differences in amplitudes of electrophysiological correlates related to empathy when shown painful pictures compared to non-painful pictures. In contrast to this, respective amplitude differences were seen in controls.

Gaming addiction studies yielded inconsistent results. Cudo et al. (2019) found that problematic video gaming correlated positively with increased IRI PD in males only, and their results also seem to be consistent with the fact that sex differences have been observed previously especially for emotional empathy with an obvious advantage for females in comparison to males (Christov-Moore et al., 2014). The other study by Mohammadi et al. (2020) did not find any differences between groups on IRI subscores and on the IRI total score. They also additionally used the emotion reactivity scale from the Scales for the assessment of emotional experience (Behr & Becker, 2004) and found that emotional reactivity was higher in the addiction group compared to controls. Finally, Stockdale and Coyne’s (2020) findings are consistent with most of the studies. They found that pathological social media use was negatively correlated with empathy.

Taken together, most consistently, the IRI PD scale discriminated between individuals with and without behavioral addictions, mostly suggesting increased PD scores in the addicted samples.

##### EQ

Collins and Freeman (2013), whose sample consisted of 73 problematic video gamers, 263 non-problematic video gamers, and 71 non-video game players, also did not find a link between behavioral addictions and empathy. These authors used the Game Addiction Scale (Lemmens et al., 2009) and the EQ to measure empathy. Their focus was on multiplayer online role-playing gamers and they concluded that although social interaction is inherent to these games, it does not necessarily mean that it also relies on empathic abilities. When comparing their results to the previously mentioned study by Cudo et al. (2019) that used different inclusion criteria and empathy measuring tools, the inconsistencies in the results are evident.

##### IRI and EQ

Another two studies compared Chinese and German samples of people with internet addiction (and one additionally focused on smartphone addiction), using both the IRI and the EQ as self-report instruments.

Lachmann et al. (2018) focused on both internet addiction, which was assessed by a short version of Young’s Internet Addiction Test (1998), and smartphone addiction, which was assessed by a short version of the Smartphone Addiction Scale (Kwon et al., 2013), in non-clinical German and Chinese samples. Two samples were investigated in this study: a sample from China, which consisted of 612 participants, and a sample from Germany, which consisted of 304 participants. The Chinese sample comprised a higher percentage of male participants compared to the German sample. The other study, by Melchers et al. (2015), was carried out in individuals with problematic internet use, also assessed by Young’s test (1998). Two samples were also investigated in this study: a sample from China which consisted of 438 participants and a sample from Germany which consisted of 202 participants, both comparable regarding the educational level. Significant associations were primarily seen in the Chinese samples compared to the German samples, although there were some differences: Lachmann et al. (2018) found that both types of addiction in both samples were associated positively with IRI PD scores, again reflecting increased PD with higher addiction scores, as in the study presented before. In contrast to this, Melchers et al. (2015) found this association only in the Chinese sample. Additionally, Lachmann et al. (2018) found that the internet addiction group showed impaired scores for EC only in the Chinese sample. On the other hand, Melchers et al. (2015) found a link between more severe addiction and deficits on the IRI EC subscale in the German sample.

As for cognitive empathy, both studies also found that increased addiction scores were associated with higher IRI FS scores (Lachmann et al. (2018): smartphone addiction/ Melchers et al. (2015): internet addiction) and lower IRI PT scores (Lachmann et al. (2018): smartphone addiction and internet addiction/ Melchers et al. (2015): internet addiction) in the Chinese sample. Lachmann et al. (2018), additionally reported that higher internet addiction scores were only associated with higher IRI FS scores in the German sample.

Concerning the EQ, Lachmann et al. (2018) used the EQ scale in the German sample only and Melchers et al. (2015) used it with both samples. Both studies again found overall lower EQ scores being related to increased scores on the assessment of behavioral addictions. Interpreting such results purely based on cultural differences must be done with caution, as comparing the two samples was not the main goal of the study by Lachmann et al. (2018). Thus, the sample size differences and some demographical differences between the two samples might have played a role. Additionally, it has to be considered that the time of emergence and the distribution of both types of behavioral addictions is different in Asia compared to Europe. Regarding the study by Melchers et al. (2015), although there were demographical differences between the samples, a main effect of country was still observed after correction for gender and age. Unfortunately, the knowledge about the association between empathy and culture is limited.

##### Other Types of Empathy Assessment

Dell’Osso et al. (2019) studied problematic internet use employing the AdAS questionnaire (Dell’Osso et al., 2017). In addition to an empathy subscale, the questionnaire also has an item that was used to assess problematic internet use. The sample consisted of 178 participants (27.5% presented with putative problematic internet use). They found that the problematic internet use group showed significantly higher empathy scores than the non-problematic internet use group. Regarding the very limited assessment of internet addiction in this study, using one item only, it is difficult to draw any specific conclusions based on this.

Taken together, a fairly consistent finding that can be derived from the four studies that assessed empathy using a self-report instrument (IRI) is that increased severity of the behavioral addictions may be related to increased personal distress, an emotional empathy dimension that usually reflects impaired self-other distinction in social interactions (Bukowski et al., 2021). Overall, the studies in this section are mainly based on self-report assessments of empathy only (except for one study: Kopiś-Posiej et al., 2021).

#### ToM

Three studies measured ToM in behavioral addictions. One used a behavior-based measure (Ünal-Aydın et al., 2020) and two studies used self-report measures (Ciccarelli et al., 2022; Cosenza et al., 2019). One of these studies focused on social media addiction, one on gambling and one on gaming.

In the study by Ünal-Aydın et al. (2020), the Reading the Mind in the Eyes Test (RMET) (Baron-Cohen et al., 2001) was used, which involves recognizing complex affective mental states, based on black and white photographs of the eye region only. The sample consisted of 317 participants divided into 120 non-addicted participants and 197 SNS addicted participants. The two subgroups did no differ in terms of gender, education, marital, economic status, or residential area and showed similar patterns in tobacco and alcohol use. Ünal-Anydin et al. (2020) found RMET deficits in individuals with SNS addiction (measured by The Social Media Addiction Scale (Tutgun-Ünal, 2015)) compared to non-addicted individuals for the RMET total and negative emotion subscores, but not for the positive or neutral subsets.

The other two studies used the Reflective Functioning Questionnaire (RFQ-8) (Fonagy et al., 2016) which measures two processes of reflective functioning: certainty about mental states and uncertainty about mental states. Cosenza et al. (2019) focused on gambling addiction which was assessed using the South Oaks Gambling Screen Revised for Adolescents (Winters et al., 1993) and their sample consisted of 410 participants (70% were classified as non-problem gamblers, 20.2% were classified as at-risk gamblers and 9.8% were classified as problem gamblers). Ciccarelli et al. (2022) focused on internet gaming addiction which was measured according to the DSM-5 diagnostic criteria using the Internet Gaming Disorder Scale—Short-Form (Pontes & Griffiths, 2015). Their sample consisted of 466 participants. Problem gamblers scored significantly higher on the RFQ-8 Uncertainty dimension, and significantly lower on the RFQ-8 Certainty subscale than non-gamblers (Cosenza et al., 2019). The other study (Ciccarelli et al., 2022) found that problematic gaming behavior was negatively correlated with Certainty, and positively correlated with Uncertainty.

Taken together, there is evidence for ToM impairment in individuals with behavioral addictions.

## General Conclusion and Future Directions

### Brief Summary of Major Findings

The present article aimed to review empirical publications that focused on different types of behavioral addictions for the following domains of sociocognitive functioning: emotion recognition, empathy/ToM.

Emotion recognition is considered a more basic construct of social cognition and the five studies that focused on emotion recognition suggest that emotion recognition is impaired in distinct samples showing behavioral addictions. The tasks used in these studies differed in nature and difficulty and did not only include facial expressions, but also used musical, vocal, and social-episodes tasks. Some only used basic emotions (or some of the basic emotions) (e.g. Chen et al., 2017b) and others focused also on more complex emotion recognition (e.g. Kornreich et al., 2016). It seems that recognizing less complex facial emotion expressions, such as happy and sad facial expressions that could be considered as unconscious emotional expressions (Peng et al., 2017) could be intact in individuals with behavioral addictions.

Impaired social cognition can affect social integration negatively in addicted patients (Volkow et al., 2011). Emotion recognition represents an important non-verbal factor in human interactions, and as Engelberg et al. (2004) mentioned in their study, being aware of emotional cues in face-to-face interactions could lead to more rewarding interactions which therefore would result in it being more preferable than any alternative interactions.

Concerning empathy, which was the most frequently studied aspect of social cognition, most studies conclude that individuals with behavioral addictions suffer from empathy impairment, especially in terms of increased personal distress. All except for one study (where an electroencephalogram paradigm was used) (Kopiś-Posiej et al., 2021) relied on self-report measures for the assessment of empathy. Some cultural differences were seen but they should be interpreted with caution as other factors mentioned above may explain the differences. Also, sex differences may play a role (Cudo et al., 2019), which is consistent with the research focusing on non-problematic gaming that showed that overall video gaming is not significantly associated with lower empathy in females (e.g. Fraser et al., 2012).

The results of two studies focusing on empathy seemingly contradicted the rest of the studies. One was by Mohammadi et al. (2020) and the other one was by Collins and Freeman (2013), both of which did not find a link between empathic abilities and behavioral addictions. As the IRI was not the main measure in the study by Mohammadi et al. (2020), the IRI-related results were not the main focus and thus were not discussed further. The main focus in the study by Collins and Freeman (2013) was on multi-player games. One can conclude that multiplayer gaming by itself is a factor that needs further research. The authors suggested that although interaction with others is present in these types of games, this might not necessarily involve empathy. The research on short term effects of multiplayer gaming (not reviewed in our paper) yielded other results. A study by Greitemeyer (2013) in which effects of team-play versus individual playing were observed concluded that as playing a violent video game cooperatively in a team, participants reported more empathy than participants who had played a violent video game on their own.

For ToM, three studies were retrieved which provide us with some evidence that individuals with behavioral addictions show some ToM impairments. The authors in these studies used behavior-based (the RMET) and self-report measures (RFQ-8) and reached the same conclusion. The RMET is sometimes interpreted as a measure of emotion recognition but mostly as a measure of cognitive empathy or ToM in the literature (Lee et al., 2021), although this notion has been challenged recently (Kittel et al., 2021). Addressing ToM during the intervention could effectively reduce behavioral addiction although in some instances, its role was mediating anxiety and perceived loneliness (Ciccarelli et al., 2022).

In general, experiencing negative effects from behavioral addictions differs from SUD in some regards, as, at least during outpatient treatment, one cannot usually stop using the internet or the mobile phone. Therefore, it is also important to study the effects of regular vs. short-term use of internet, video games, SNS, mobile phones. Some studies found a positive correlation with different aspects of social cognition (Smith et al., 2020) and other studies found negative correlations (Fraser et al., 2012; Miedzobrodzka et al., 2021). Studying non-problematic, i.e. clinically not-relevant gaming, gambling, SNS or internet use can guide further research which focuses on behavioral addictions, especially when taking into account other factors that might affect different aspects of social cognition. Factors can include game type play, as being exposed to in-game storytelling enhances affective ToM (Bormann & Greitemeyer, 2015). The played character can also be taken into account as total playtime as a support character was positively related to social cognition (Delhove & Greitemeyer, 2020). Players with preexisting social networks and social skills can potentially benefit from interaction on online platforms (Castillo, 2019). Taking these aspects into consideration can be used a guidance for further research which focuses on behavioral addictions.

### Limitations of Current Research and Directions for Future Research

The interest in social cognition has increased in the past 10 years, but the literature focusing on behavioral addictions is still scarce and insufficient to reach solid conclusions. This can be attributed to different reasons, most of which refer to methodology. First and foremost, it is crucial to identify the relevant sociocognitive constructs, and based on that to choose the best measures which are suitable to sensitively assess these aspects of interest. As discussed previously, in the studies focusing on ToM, the RMET was initially used to assess facial emotion recognition skills. Choosing the correct tool can help to identify the exact pattern of deficits which is crucial for planning interventions.

Another problem concerning sociocognitive assessment is that almost all studies investigating empathy and ToM relied on self-report measures. Only for emotion recognition and in only one study assessing empathy (Kopiś-Posiej et al., 2021) and in one study assessing ToM (Ünal-Aydın et al., 2020), performance-based measures were used. Self-reported abilities may not actually represent the actual level of the ability itself, especially when dealing with potential impairments of sociocognitive functions such as empathy and ToM, as they rely on a certain level of metacognitive awareness in the affected individual to be reported (Murphy & Lilienfeld, 2019).

Although gambling is the only disorder for which diagnostic criteria have been specified in the DSM-5, there is some consistency in assessing internet addiction across studies (Young, 1998). Other than that, only one study clinically confirmed diagnosis in their sample by a psychiatrist alongside using an addiction questionnaire (Fan et al., 2022). Nevertheless, lack of diagnostic criteria for some behavioral addiction subtypes can lead to different inclusion criteria for the samples assessed across studies and thus contribute to inconsistencies in the results, see e.g., for the two studies focusing on empathy in gamers (Cudo et al., 2019; Mohammadi et al., 2020). Additionally, including a matched control comparison group (e.g. Kornreich et al., 2016) affects the reliability of the results, and could make it easier to compare results between the studies and to reach overall conclusions.

Finally, measuring the overall neurocognitive functioning of the included participants is also an important point that has not been considered in the currently reviewed literature, although general cognitive performance is clearly related to social cognition (Channon, 2004; Thoma et al., 2013). Assessing cognitive functions in future studies can elucidate whether the sociocognitive deficits are related to the cognitive impairments. Finally, comorbidity is common in behavioral addictions, e.g., for pathological gamblers and Personality Disorders (Ibáñez et al., 2001). Thus, taking comorbidities into account when implementing studies (inclusion criteria for samples) is important.

The present review is also characterized by a couple of limitations. First, only studies that focused on adult populations (≥ 18 years of age) were included as the adolescent samples in the potentially retrieved studies were very heterogeneous. Thus, reaching a meaningful conclusion about the association between sociocognitive impairment and behavioral addictions would have been difficult. One further reason why 18 + was chosen as a cut off age is because during late adolescence (18–20 years of age) both behavioral addictions and personal distress related to these seem to decrease significantly (Verrastro et al., 2021).

Second, not all sociocognitive functions were included in the present review. Although emotional and cognitive empathy as well as ToM can be considered as essential building blocks for social skills and social problem solving (Pertz et al., 2020; Thoma et al., 2013), social skills represents a very broad term which encompasses several abilities that help to initiate, maintain and reach desired goals during interaction with others (Morgan, 1980). This can include several verbal/non-verbal skills and can be as basic as eye contact or more complex skills such as social decision making (Jurevičiene et al., 2018) rendering this construct very diverse.

## Overall Conclusions

The results of the majority of the retrieved studies on the relationship between distinct sociocognitive aspects and behavioral addictions allow for the conclusion that individuals with behavioral addictions exhibit some social cognitive deficits. Other than the low number of studies, some major methodological issues concerning assessment of both behavioral addictions and sociocognitive functioning should be taken into account in future research. At this point, conclusions should be drawn with caution concerning social cognition in populations presenting with behavioral addictions. Also, the effects of the current Covid-19 pandemic and how, related to this, increased use of media and internet might affect our mental health (Holmes et al., 2020) and the development of addictions, are not clear. By taking into account the issues mentioned in the previous section, future research can lead to a better understanding of these constructs in individuals with behavioral addictions, which can lead to higher awareness and better intervention planning.

## Supplementary Information

Below is the link to the electronic supplementary material.

Supplementary file1 (PDF 83 KB)

Supplementary file2 (PDF 148 KB)

## Data Availability

The data supporting the results are available within the current review.
