# Supplementary material for: Impairments of Sociocognitive Functions in Individuals with Behavioral Addictions: A Review Article
Source: J Gambl Stud. 2023 Jun 12;40(2):429–51. doi: 10.1007/s10899-023-10227-w (PMC10259812; doi:10.1007/s10899-023-10227-w)
Supplement: Supplementary file 1 — Supplementary file1 (PDF 83 KB) [file 10899_2023_10227_MOESM1_ESM.pdf]

Impairments of sociocognitive functions in individuals with behavioral addictions: A review article

Journal of Gambling Studies

Dalia Arafat[0000-0001-9722-6919]*, Patrizia Thoma[0000-0003-4415-1719]
Neuropsychological Therapy Centre
Faculty of Psychology, Ruhr University of Bochum
Universitätsstraße 150, 44801 Bochum, Germany

*Corresponding author's Email: dalia.arafat@ruhr-uni-bochum.de

Box *Syntax of applied search criteria in a) PubMed and b) Web of Science comprising two thematic categories: and terms associated with social cognition.*

a) ((gambl*[Title/Abstract]) OR (gaming[Title/Abstract]) OR (game[Title/Abstract]) OR (internet[Title/Abstract]) OR (social network sites[Title/Abstract]) OR (social media[Title/Abstract]) OR (SNS[Title/Abstract])) AND ((social cognition[Title/Abstract]) OR (empat*[Title/Abstract]) OR (ToM[Title/Abstract]) OR (theory of mind[Title/Abstract]) OR (mentalizing[Title/Abstract]) OR (social skills[Title/Abstract]) OR (social problem solving[Title/Abstract]) OR (emotion recognition[Title/Abstract]) OR (affect recognition[Title/Abstract]))

b) (AB=(((gambl*) OR (gaming) OR (game) OR (internet) OR (social network sites) OR (social media) OR (SNS)) AND ((social cognition) OR (empat*) OR (ToM) OR (theory of mind) OR (mentalizing) OR (social skills) OR (social problem solving) OR (emotion recognition) OR (affect recognition)))) OR TI=(((gambl*) OR (gaming) OR (game) OR (internet) OR (social network sites) OR (social media) OR (SNS)) AND ((social cognition) OR (empat*) OR (ToM) OR (theory of mind) OR (mentalizing) OR (social skills) OR (social problem solving) OR (emotion recognition) OR (affect recognition)))
