# Supplementary material for: Impairments of Sociocognitive Functions in Individuals with Behavioral Addictions: A Review Article
Source: J Gambl Stud. 2023 Jun 12;40(2):429–51. doi: 10.1007/s10899-023-10227-w (PMC10259812; doi:10.1007/s10899-023-10227-w)
Supplement: Supplementary file 2 — Supplementary file2 (PDF 148 KB) [file 10899_2023_10227_MOESM2_ESM.pdf]

Impairments of sociocognitive functions in individuals with behavioral addictions: A review article

Journal of Gambling Studies

Dalia Arafat[0000-0001-9722-6919]*, Patrizia Thoma[0000-0003-4415-1719]
Neuropsychological Therapy Centre
Faculty of Psychology, Ruhr University of Bochum
Universitätsstraße 150, 44801 Bochum, Germany

*Corresponding author's Email: dalia.arafat@ruhr-uni-bochum.de

Table 1: Overview of studies included in the review summarizing methodology and key findings in chronological order.

Number	Authors	Addiction	Time of assessment	Participants	Sociocognitive Tasks	Key findings on sociocognitive functions for the patients relative to the respective control groups
1	Engelberg et al., 2004	internet	Current users	41 participants	Emotion identification task (facial expressions and social episodes)	Negative relationship between internet addiction and both emotion recognition tasks.

2	Collins & Freeman et al., 2013	Video gaming	Current gamers	73 problematic video gamers, 263 non-problematic video gamers, 71 non-video game players	EQ	No group differences
3	Melchers et al., 2015	Internet	Current users	Two samples: 438 from China and 202 from Germany, same educational level in both groups	IRI, EQ	Low general empathy (low scores on the EQ) is associated with problematic internet use in both samples<br>Specific associations between IRI and problematic internet use (see section 2.2)

Table 1: (Continued)

Number	Authors	Addiction	Time of assessment	Participants	Sociocognitive Tasks	Key findings on socio-cognitive functions for the patients relative to the respective control groups
4	Kornreich et al., 2016	Gambling	Patients seeking ambulatory treatment	22 male participants with pathological gambling, 22 controls matched for sex, age and education level	Musical (happiness, sadness, threat, peacefulness) vocal (anger, disgust, fear, sadness, surprise, and happiness, in addition to a neutral condition) and facial (happiness, sadness, fear, anger and 5 neutral faces) emotion recognition task	Some differences in vocal and facial emotion recognition (see section 2.1)
5	Chen et al., 2017	Internet	Current users	97 participants: 54% no-Internet addiction symptom group, 41% mild-Internet addiction symptom group, and 5% severe-Internet addiction symptom group	Validated computerized emotion recognition task (anger, disgust, fear, happiness, sadness, surprise)	Positive relationship between deficits in recognizing disgust facial expression only and Internet addiction

Table 1: (Continued)

Number	Authors	Addiction	Time of assessment	Participants	Sociocognitive Tasks	Key findings on sociocognitive functions for the patients relative to the respective control groups
6	Jiao et al., 2017	Internet	Current users	16 participants in the internet addiction group, 16 healthy controls	IRI	Differences only on the IRI PD scale between the two groups
7	Tomei et al., 2017	Gambling	Outpatients	31 non-gamblers, 24 healthy gamblers, 21 problem gamblers, Problem Gambling Severity Index, unmatched on several variables prior to the assessments	IRI	Significant differences between the Problem Gamblers group and the other two groups together with regard to IRI FS, PT, and PD, but not EC
8	Peng et al., 2017	Internet gaming	Currents gamers	16 gamers, 16 controls. No significant differences in age, handedness, and education.	Computerized emotion recognition task (sad, happy, neutral)	Slower reaction times in recognizing sad faces in gamers when compared to controls.
9	Cosenza et al., 2018	Gambling	Current gamblers	410 participants: 70% of participants were classified as non-problem gamblers, 20.2% as at-risk gamblers, and 9.8% as problem gamblers	RFQ-8	Higher RFQ-8 Uncertainty scores, and lower RFQ-8 Certainty scores in the problem gamblers group compared to non-gamblers.

Table 1: (Continued)

Number	Authors	Addiction	Time of assessment	Participants	Sociocognitive Tasks	Key findings on sociocognitive functions for the patients relative to the respective control groups
10	Lachmann et al., 2018	Internet and smartphone	Current users	Two samples: 612 from China and 304 from Germany, gender differed significantly across the two samples	IRI, EQ	Low general empathy (low scores on the EQ) is associated with problematic internet use in German Sample<br>Specific associations between IRI and problematic internet use (see page 12)
11	Cudo et al., 2019	Video gaming	Current gamers	370 video gamers (169 male gamers)	IRI	A positive correlation between IRI PD and problematic video gaming in male gamers
12	Dell'Osso ety al., 2019	Internet	Current users	178 participants; 27,5% presented putative problematic internet use	Empathy subscale (The AdAS Spectrum questionnaire)	Higher empathy scores in problematic internet use group compared to the non-problematic internet use group

Table 1: (Continued)

Number	Authors	Addiction	Time of assessment	Participants	Sociocognitive Tasks	Key findings on sociocognitive functions for the patients relative to the respective control groups
13	Mohammadi et al. (2020)	Gaming	Current gamers	29 gamers playing violent videogames and 29 controls, matched with age, sex, school education, handedness	IRI and emotional reactivity scale	IRI subscores and total score did not differ between groups. Higher emotional reactivity in addiction group relative to controls
14	Stockdale and Coyne 2020	Social media	Current users	385 participants	IRI	Pathological social media use was negatively corelated with empathy
15	Ünal-Aydın et al., 2020	SNS	Current internet users	317 participants divided into: 120 non-addicted participants, 197 SNS addicted participants: no differences in gender, education, marital, economic status, residential area, and similar patterns in tobacco and alcohol use	RMET	Deficits in SNS addiction individuals on RMET-negative subtest and RMET-total

Table 1: (Continued)

Number	Authors	Addiction	Time of assessment	Participants	Sociocognitive Tasks	Key findings on sociocognitive functions for the patients relative to the respective control groups
16	Kopi´s-Posiej et al., 2021	Facebook	Current users	21 participants in the low problematic Facebook use group and 22 participants in the high problematic Facebook use group	Empathy for pain task	Longer response time to Facebook related stimuli in the High problematic Facebook use group<br>No differences between the group in terms of rating the stimuli.
17	Ciccarelli et al., 2022	Gaming	Current gamers	466 participant	RFQ-8	Negative correlation between problematic gaming behavior and Certainty. Positive correlations between problematic gaming and Uncertainty
18	Fan et al., 2022	Gaming	Current gamers	60 participants with internet gaming disorder and 60 controls	Emotion recognition task (happy, neutral and angry faces)	Worse performance of the addiction group in terms of accuracy reaction time (see section 2.1).

Key: AdAS — The Adult Autism Subthreshold Spectrum; EC — Empathic Concern; EQ — Empathy Quotient; FS — Fantasy; IRI — Interpersonal Reactivity Index; PD — Personal Distress; PT — Perspective Taking; RFQ — the Reflective Functioning Questionnaire; RMET — Reading the Mind in the Eyes Test; SNS — Social networking site.

References

References marked with an asterisk indicate studies included in the review.

Chen, Z., Poon, K.-T., & Cheng, C. (2017). Deficits in recognizing disgust facial expressions and internet addiction: Perceived stress as a mediator. *Psychiatry research*, 254, 211–217. https://doi.org/10.1016/j.psychres.2017.04.057

Ciccarelli, M., Nigro, G., D'Olimpio, F., Griffiths, M. D., Sacco, M., Pizzini, B., & Cosenza, M. (2022). The associations between loneliness, anxiety, and problematic gaming behavior during the covid-19 pandemic: The mediating role of mentalization. *Mediterranean Journal of Clinical Psychology*, 10 (1).

Collins, E., & Freeman, J. (2013). Do problematic and non-problematic video game players differ in extraversion, trait empathy, social capital and prosocial tendencies? *Computers in Human Behavior*, 29 (5), 1933–1940. https://doi.org/10.1016/j.chb.2013.03.002

Cosenza, M., Ciccarelli, M., & Nigro, G. (2019). The steamy mirror of adolescent gamblers: Mentalization, impulsivity, and time horizon. *Addictive Behaviors*, 89, 156–162.

Cudo, A., Kopiś, N., & Zabielska-Mendyk, E. (2019). Personal distress as a mediator between self-esteem, self-efficacy, loneliness and problematic video gaming in female and male emerging adult gamers. *PLOS ONE*, 14 (12), e0226213. https://doi.org/10.1371/journal.pone.0226213

Dell'Osso, L., Bertelloni, C., Di Paolo, M., Avella, M., Carpita, B., Gori, F., Pompili, M., & Carmassi, C. (2019). Problematic internet use in university students attending three superior graduate schools in italy: Is autism spectrum related to suicide risk? *International Journal of Environmental Research and Public Health*, 16, 1098. https://doi.org/10.3390/ijerph16071098

Engelberg, E., & Sjöberg, L. (2004). Internet use, social skills, and adjustment. *Cyberpsychology & behavior*, 7 (1), 41–47.

Fan, L., He, J., Zheng, Y., Nie, Y., Chen, T., & Zhang, H. (2022). Facial micro-expression recognition impairment and its relationship with social anxiety in internet gaming disorder. *Current Psychology*, 1–10.

Jiao, C., Wang, T., Peng, X., & Cui, F. (2017). Impaired empathy processing in individuals with internet addiction disorder: An event-related potential study. *Frontiers in Human Neuroscience*, 11, 498.

Kopiś-Posiej, N., Cudo, A., Tużnik, P., Wojtasiński, M., Augustynowicz, P., Zabielska-Mendyk, E., & Bogucka, V. (2021). The impact of problematic facebook use and facebook context on empathy for pain processing: An event-related potential study. *Computers in Human Behavior*, 124, 106936.

Kornreich, C., Saeremans, M., Delwarte, J., Noël, X., Campanella, S., Verbanck, P., Ermer, E., & Brevers, D. (2016). Impaired non-verbal emotion processing in pathological gamblers. *Psychiatry research*, 236, 125–129. https://doi.org/10.1016/j.psychres.2015.12.020

Lachmann, B., Sindermann, C., Sariyska, R. Y., Luo, R., Melchers, M. C., Becker, B., Cooper, A. J., & Montag, C. (2018). The role of empathy and life satisfaction in internet and

smartphone use disorder. *Frontiers in psychology*, 9, 398. https://doi.org/10.3389/fpsyg.2018.00398

Melchers, M., Li, M., Chen, Y., Zhang, W., & Montag, C. (2015). Low empathy is associated with problematic use of the internet: Empirical evidence from china and germany. *Asian Journal of Psychiatry*, 17, 56–60. https://doi.org/10.1016/j.ajp.2015.06.019

Mohammadi, B., Szycik, G. R., Te Wildt, B., Heldmann, M., Samii, A., & Münte, T. F. (2020). Structural brain changes in young males addicted to video-gaming. *Brain and cognition*, 139, 105518. https://doi.org/10.1016/j.bandc.2020.105518

Peng, X., Cui, F., Wang, T., & Jiao, C. (2017). Unconscious processing of facial expressions in individuals with internet gaming disorder. *Frontiers in Psychology*, 8, 1059.

Stockdale, L. A., & Coyne, S. M. (2020). Bored and online: Reasons for using social media, problematic social networking site use, and behavioral outcomes across the transition from adolescence to emerging adulthood. *Journal of adolescence*, 79, 173–183.

Tomei, A., Besson, J., & Grivel, J. (2017). Linking empathy to visuospatial perspective-taking in gambling addiction. *Psychiatry research*, 250, 177–184. https://doi.org/10.1016/j.psychres.2016.12.061

Ünal-Aydın, P., Balıkçı, K., Sönmez, İ., & Aydın, O. (2020). Associations between emotion recognition and social networking site addiction. *Psychiatry research*, 284, 112673. https://doi.org/10.1016/j.psychres.2019.112673
